# Estimating the burden of leptospirosis in Sri Lanka; a systematic review

**DOI:** 10.1186/s12879-018-3655-y

**Published:** 2019-02-06

**Authors:** Janith Warnasekara, Iresha Koralegedara, Suneth Agampodi

**Affiliations:** Department of Community Medicine, Faculty of Medicine and Allied Sciences, Rajarata University of Sri Lanka, Saliyapura, 50008 Sri Lanka

## Abstract

**Background:**

Although the assessment of disease burden should be a priority for allocating resources, leptospirosis is grossly underestimated despite its true burden in Sri Lanka. This study aimed to assess the morbidity and mortality of leptospirosis based on routine surveillance data, hospital reported data and scientific publications from Sri Lanka.

**Method:**

A systematic review was carried out, and Pub Med, MEDLINE®, BIOSIS Previews, Zoological Record, Web of Science Core Collection, Current Contents Connect, KCI-Korean Journal Database, BIOSIS Citation Index, Data Citation Index, SciELO Citation Index and Google Scholar databases were searched. Quarterly epidemiological bulletin (QEB), indoor morbidity & mortality returns (IMMR) and hand searches of local literature were performed in local libraries. Forty-two relevant full texts, 32 QEBs, and 8 IMMR were included in the full text review. Adjustments were made for under diagnosis, underreporting and chance variability.

**Results:**

The estimated annual caseload of leptospirosis in Sri Lanka from 2008 to 2015, was 10,423, and the cumulative annual incidence of leptospirosis that required hospitalization was 52.1 (95% CI 51.7–52.6) per 100,000 people. The estimated number of annual deaths due to leptospirosis was approximately 730 (95% CI 542–980), with an estimated pooled case fatality ratio of 7.0% (95% CI 5.2–9.4). The most common organs involved were the kidney, liver and heart, with median rates of 48.7, 30, and 14.2%, respectively.

**Conclusion:**

Our systematic review shows gross underestimation of the true leptospirosis burden in the national statistics of Sri Lanka, and the hospitalization rates estimated in our study were compatible with the total burden estimate of 300·6 (95% CI 96·54–604·23) per 100,000 people published previously.

**Electronic supplementary material:**

The online version of this article (10.1186/s12879-018-3655-y) contains supplementary material, which is available to authorized users.

## Background

Leptospirosis accounts for an estimated 2.9 million disability adjusted life years (DALYs) annually [1] due to an average of 1.03 million cases and 58,900 deaths [2]. The disease is caused by 11 pathogenic and 5 intermediate species of *Leptospira* from the genus *Leptospira* of the family Leptospiraceae [3]. Large numbers of hosts, such as livestock, domestic pets, wild or feral animals, excrete *Leptospira* from their proximal renal tubules, and humans are infected through direct or indirect contact with the infected urine of those hosts through abrasions of the skin, mucus membranes or conjunctiva. Due to the wide variety of hosts available for transmission and the facilitating environmental conditions, *Leptospira* has one of the widest geographical distributions among zoonotic diseases [4].

Clinically, leptospirosis can present from mild flu-like illness to severe life threatening systematic manifestations, such as pulmonary haemorrhages, acute kidney injury, myocarditis, pancreatitis or multi organ dysfunction syndrome (MODS) [5]. Leptospirosis is recognized as one of the causes of pyrexia of unknown origin or undifferentiated fevers [6]. As a result, there are many unreported cases of leptospirosis classified as undifferentiated fevers. Despite all the reported complications, the complication rates are yet to be established. Unawareness of complications will lead to underdiagnosis and therefore the poor prediction of outcomes. Leptospirosis mimics dengue, hantavirus, malaria, rickettsioses and viral sepsis [7, 8] which can cause delayed diagnosis and increased mortality. Although the microscopic agglutination test (MAT) was considered “standard” for diagnosing leptospirosis [9], it is no longer considered the “gold standard” due to its well documented low sensitivity and predictive values [9, 10]. Lack of point of care diagnostic facility severely affect the leptospirosis diagnosis; hence, the global disease estimates may not be entirely valid for country level estimates.

Sri Lanka has experienced large and frequent outbreaks of leptospirosis during the last decade [11]. In 1959, *Leptospira* was first isolated from the blood of a patient in Colombo [12]. Since then, cases have been reported from almost all regions in Sri Lanka. In 2008, 7421 cases of leptospirosis were notified to the epidemiology unit of Sri Lanka. Since 2008, the Sri Lankan disease surveillance programme has received large numbers of annual notifications of suspected cases of leptospirosis, and Sri Lanka is globally considered to have one of the highest incidences of leptospirosis. However, the exact number is uncertain due to the lack of definitive diagnostics and deficiencies of the notification system [13]. Both physicians and epidemiologists often question the data from the routine notification system due to inadequate reporting.

In 2015, Paul R. Torgerson et al [1] and a systematic review by Federico Costa et al. [2] provided estimates on the global disease burden. However, they highlighted the absence of local data as a major barrier to true global disease estimates. Hence, this Sri Lankan case study will help to understand the morbidity and mortality of leptospirosis in other tropical countries, where the disease burden remains unknown. Although leptospirosis is currently considered one of the most important communicable diseases in Sri Lanka, disease incidence estimates have not been done beyond routinely published data from the national surveillance centre. This systematic review aimed to describe the morbidity and mortality due to leptospirosis in Sri Lanka using different sources of data in order to better understand the problem.

## Methods

We performed a comprehensive literature search to identify studies and grey literature related to morbidity and mortality of leptospirosis in Sri Lanka. The study protocol (Additional file 1) was prepared according to the guidelines of the Cochrane collaboration [14]

### Eligibility criteria

We recruited studies and reports containing details of possible cases of human leptospirosis originating from Sri Lanka between 1900 and 2017. All types of publications with primary data were included, and no language restrictions were employed. Both published and unpublished research and reports were included.

### Databases and information resources

For this review, we performed an Internet-based search using Pub Med, MEDLINE®, BIOSIS Previews, Zoological Record, Web of Science Core Collection, Current Contents Connect, KCI-Korean Journal Database, BIOSIS Citation Index, Data Citation Index and SciELO Citation Index. Google Scholar was used to gather all Internet-based literature not indexed in the above databases because most of the local journals are not indexed in the selected databases, and it is common to have technical reports (usually available in Google Scholar) rather than journal articles in the Sri Lankan setup. In addition, we used four bibliographic references to search for local literature: Bibliography of medical publications related to Sri Lanka 1811–1976 [15] and its supplement Bibliography on health in Sri Lanka, 1977–1980 [16] by Peiris and Uragoda; Bibliography of Medical Literature 1980–2005 compiled by the Post Graduate Institute of Medicine (PGIM) Library, Colombo; and the Annotated Bibliography of dissertations and theses Presented to PGIM by Postgraduate Trainees, published by the PGIM. Further, we searched technical reports published by the Medical Research Institute (MRI), papers published on the Ceylon Medical Journal before 2008 (the CMJ website is available after 2008), archived issues of the Sri Lanka Journal of Medical Sciences and the Kandy Medical Journal in four libraries (Sri Lanka Medical Association Library, PGIM library and Colombo and Peradeniya Medical faculty libraries). Throughout the process, we searched reference lists of selected articles to include missing articles from the main search. For national level data, we used several sources. Quarterly epidemiological bulletin (QEB) data of the epidemiology unit of Sri Lanka and the data published by the epidemiology unit in disease trends (http://www.epid.gov.lk) were compared to obtain the best data set for national “surveillance” data. These surveillance data were from routine notifications from hospitalized patients and outpatients and were considered more “representative” of all cases. The Indoor Morbidity and Mortality Returns (IMMR) available from the Ministry of Health were obtained as “hospital” data. IMMR data are based on diagnoses made during the clinical management at an inward setup. This diagnosis is typically based on clinical criteria and varies widely from physician to physician. IMMR data are limited to hospitalized patients and may represent only moderate to severe cases, as mild patients are treated as outpatients.

### Search strategies

The key themes for the search included “Leptospirosis” or “Leptospira” in combination with “Sri Lanka” or “Ceylon”. For the PubMed search, appropriate search terms were identified and translated to *MeSH* terms where possible. Entry items and keywords used in the initial search related to “Leptospira” are listed in Additional file 2. The PubMed search string used was (“Leptospira”[Title/Abstract]. OR “Leptospirosis”[Title/Abstract]. OR “Weil Disease”[Title/Abstract]. OR “Leptospirosis”[Mesh]. OR “Weil Disease”[Mesh]. OR “Leptospira”[Mesh]) AND (“Ceylon” OR “Sri Lanka”). Other databases were searched through Web of Science, and the search string was TOPIC: (Leptospirosis) *OR* TOPIC: (Leptospira) *OR* TOPIC: (Weil’s disease) Refined by: TOPIC: (“Sri Lanka” OR “Ceylon”). The Google Scholar search string used was (“Leptospira”OR “Leptospirosis”OR “Weil’s Disease”) AND (“Ceylon” OR “Sri Lanka”).

### Study selection

After removal of duplicates from different databases, the title and abstract were screened to exclude articles that were not relevant. These included articles that were not directly related to leptospirosis, animal studies and articles without primary data. Conference publications were included if the data were not later published as journal articles or technical papers. Full text reviews were performed for all screen-selected articles to further assess the eligibility criteria. Articles reporting diagnosed cases of leptospirosis and deaths due to leptospirosis were included in the final evidence synthesis. For national disease estimates, we included reports with possible cases of leptospirosis.

### Data collection process

The data were extracted using an Excel data sheet by two independent investigators (JW and IK), and the extracted dataset was fully reviewed for confirmation by the SA.

### Data items

Data items included in this review were used to assess the study and to describe sequelae. These included: study year, authors, duration, study population, sampling procedure, sample size (n), laboratory confirmation, complications and deaths.

### Risk of bias

Risk of bias in individual studies was assessed using a checklist (Additional file 3). The main risk of bias assessed was due to the study population. First, we searched the methods to see whether measures were taken to include the full study population. Although different study populations were allowed in this review, we assessed whether systematic inclusion of all leptospirosis patients was done either through population screening (population-based studies) or through febrile patient screening. The risk of selection bias was classified as mild, moderate or high based on this assessment. Bias due to misclassification was assessed by examining the diagnostic criteria. If the diagnosis was confirmed using the laboratory criteria given by the leptospirosis burden epidemiology research group (LERG), it was considered a study with a low risk of bias. Studies with positive screening tests but that were not confirmed as “low risk” were considered “moderate risk”, and studies without laboratory confirmation were considered at “high risk” for bias. Most of the patients recruited to the studies were moderate to severe patients. This resulted in an over estimate of the complication and mortality rates, and there were higher numbers of publications from areas where higher number of researchers were conducting studies. Hence, the risk of bias across studies due to “publication bias” or selective reporting is discussed in the results section.

### Data synthesis and estimations

We summarized the data into two main categories: national disease estimates and complications/deaths reported in studies. Construction of 95% confidence limits for observed rates and counts was performed using the Poisson distribution model using the formula $C\pm {Z}_{1-\propto /2}\sqrt{C}$ for counts and $R\pm {Z}_{1-\propto /2}\sqrt{\frac{\overline{C}}{n}}$for rates. Since hospital reported data grossly underestimate the leptospirosis incidence, we added an adjustment factor for disease incidence estimates using published research studies [17]. For this purpose, we searched for studies reporting systematic patient recruitment by screening all febrile patients (low risk for selection bias) together with disaggregated data by type of diagnosis (clinically suspected or not). This was slightly different from the global disease burden estimates that used a robust method of adjustment for underdiagnosis using multicounty data and modelling, which may not be directly applicable for in-country estimation. For Sri Lankan data, the adjustments were done conservatively using the reported under-diagnosed proportion and calculating the 95% confidence intervals for the proportion using the original data. Using these estimated proportions, we calculated high and low estimates for the caseload in each year. For case fatality rate calculations, we used studies with more than 100 confirmed cases (to reduce the chance of error), in which data were collected in defined settings over a period of at least 3 months. We calculated the pooled case fatality rate with 95% CI using the Poisson distribution model.

## Results

**Fig. 1:**
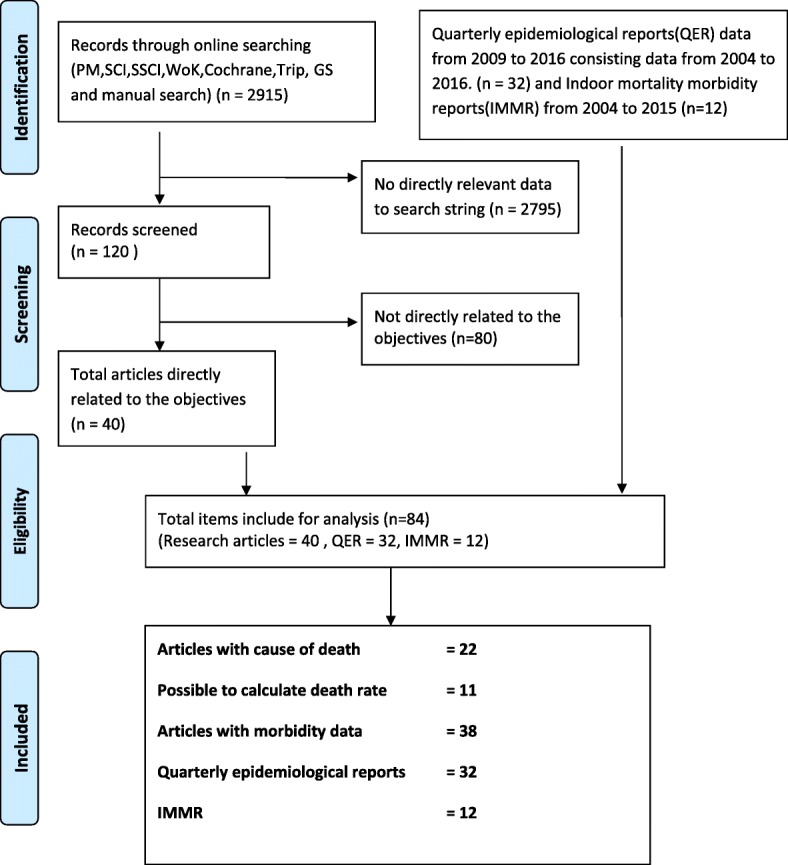
PRISMA flow diagram

The initial identification of articles and databases included 2960 items **(PRISMA flow diagram-**Fig. 1**)**. After the screening, 165 articles were assessed for eligibility, and 84 articles were included in the qualitative synthesis. We identified 42 original research papers/reports with details on sequelae and causes of death. There were 32 QEB from 2009 to 2016, and data from 2004 to 2008 were reported in the 1st quarter of 2009. After comparing the QEB data with the final dataset published by the Epidemiology unit, we used “Leptospirosis trend” data from 2010 to 2015 and QEB data from 2005 to 2009 as “surveillance data” for disease incidence estimates. IMMR consisted of reported numbers of leptospirosis patients from 2004 to 2015.

### National disease incidence estimate

There were no nationally representative incidence studies, community-based incidence studies or prospective cohort studies that could be used for national disease incidence estimates. Only two passive surveillance databases were available for this purpose: notification data (leptospirosis surveillance data) from the epidemiology unit and hospital admission data from IMMR. Surveillance data grossly underestimated the disease incidence and deaths. From 2004 to 2015, 87,075 patients were registered in IMMR as having leptospirosis, compared to 45,316 reported in the surveillance system. Almost half (*n* = 41,781, 47.0%) of the hospitalized patients were not included in the surveillance system (Table 1). For deaths, the difference was much larger, with 937 deaths reported during the study period in surveillance data compared to 2600 deaths in hospital data; nearly two thirds of deaths were missing (63.0%). The reported case fatality rate was 2.1% in surveillance data and 3.0% in hospital data.

**Table 1:** Reported number of leptospirosis cases and deaths in Sri Lanka from 2004 to 2015 based on two different data sources

Year	Reported number of leptospirosis cases		Reported number of leptospirosis deaths	
	Surveillance data	Hospital data (IMMR)	Surveillance data	Hospital data (IMMR)
2004	2243	3291	16	138
2005	1147	3900	33	147
2006	1550	3428	40	158
2007	2198	3856	34	180
2008	7421	10,051	207	357
2009	4968	8432	141	275
2010	4554	9398	122	260
2011	6689	13,104	97	265
2012	3690	6178	52	167
2013	4308	8296	81	217
2014	3235	7369	42	172
2015	4435	9772	72	264

The reasons for increased incidence after 2008 are not well understood. However, we believe that the increased awareness among treating physicians after the outbreak in 2008 contributed to enhanced reporting in subsequent years.

### Estimated caseload, disease incidence and deaths due to leptospirosis

**Fig. 2:**
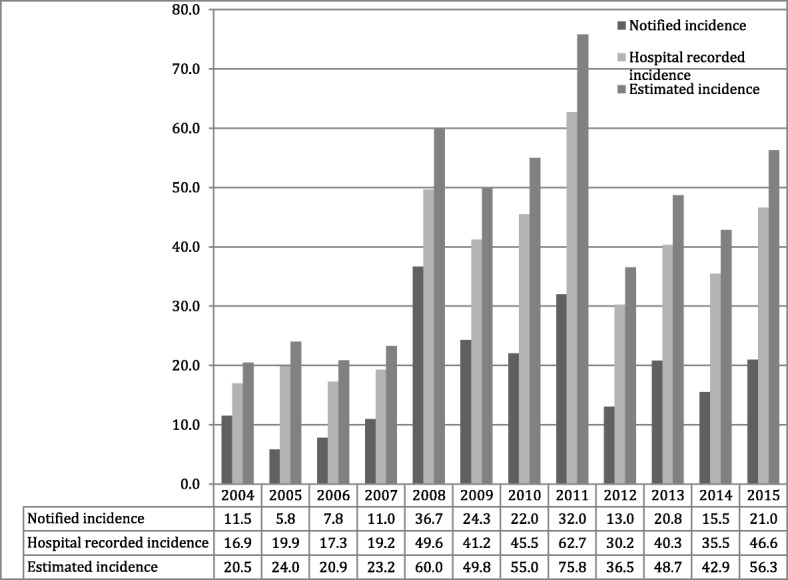
Incidence of leptospirosis in Sri Lanka: comparison of surveillance data, hospital data and estimated incidence

Data from a single study were available to calculate the hospital underestimation of cases. In the study in Kegalle in 2008, 26 probable cases fulfilling the surveillance criteria were treated as other diseases (hence not included in IMMR), while 175 patients were documented as having leptospirosis during the same period [17]. We used these numbers to estimate the annual caseload with the upper limit of 95% confidence interval for the estimates. After the adjustments, the estimated annual caseload of leptospirosis was 10,423, and the cumulative incidence of leptospirosis that required hospitalization in Sri Lanka during 2008–15 was 52.12 (95% CI 51.69–52.57) per 100,000 people per year (Fig. 2).

Even though leptospirosis was declared a notifiable disease in 1991, major attention was paid to leptospirosis after the heavy outbreak reported in 2008 [18]. Deaths due to leptospirosis were reported in surveillance reports only after this outbreak. However, the number of deaths due to leptospirosis from 2004 to 2008 was available in QEB of the 1st quarter in 2009. Neither surveillance data nor hospital data were based on confirmed cases of leptospirosis but rather were based on suspected patients of leptospirosis. Applying the reported case fatality rate from hospitals to the estimated caseload, the estimated number of annual hospital deaths due to leptospirosis was approximately 311 (95% CI 277–347) (Fig. 2).

### Complications and organ involvement

Multisystemic complications have been reported due to leptospirosis since the first report in 1959. However, a lack of consistency in reporting complications has made it difficult to estimate the true proportions. The definitions used are neither standard nor complete in most studies. Hence, the classification was done as organ “involvement” rather than specific complications. We calculated the reported rates of complications considering the total confirmed cases as the denominator. Table 2 summarizes the complications reported from Sri Lankan patients.

**Table 2:** Reported leptospirosis complications

Complication	Published Year	Citation	Method of Confirmation	Number of patients	Total patients	Percentage
Renal involvement	1964 1966 1970 1974 1976 2008 2008 2011 2011 2011 2012 2012 2013 2013 2013 2014 2014 2014 2015 2015 2015 2015 2015 2016 2016 2016 2016 2016 2016 2016 2017	[32] [33] [34] [21] [35] [36] [37] [38] [39] [20] [40] [41] [42] [43] [44] [45] [46] [29] [47] [22] [48] [49] [50] [19] [9] [51] [52] [53] [54] [55] [56]	Biochemical Biochemical Biochemical Biochemical Biochemical Biochemical Biochemical Biochemical Biochemical Biochemical Biochemical Autopsy Biochemical Biochemical Biochemical N/M Biochemical Biochemical Biochemical Autopsy Biochemical Proteinuria Biochemical Biochemical Biochemical Biochemical Biochemical Biochemical Biochemical Biochemical Biochemical	44 13 C/S 41 34 11 C/R 23 C/S 62 10 ^a^ N/M C/R 12 N/M ^a^ 7 C/R C/R 157 14 N/M 25 10 N/M C/S 60 213 113 ^a^	60 104 C/S 81 61 45 C/R 155 C/S 132 62 ^a^ N/M C/R 22 N/M ^a^ 32 C/R C/R 232 19 N/M 45 48 66 C/S 110 563 221 ^a^	73.3 12.5 C/S 50.6 55.9 24.4 C/R 14.8 C/S 46.9 16.1 ^a^ N/M C/R 54.5 30.0 ^a^ 21.8 C/R C/R 67.7 73.3 7.14 55.6 20.8 68.2 C/S 54.5 37.8 51.1 ^a^
Liver involvement	1964 1966 1967 1974 1976 2003 2011 2011 2011 2011 2012 2013 2013 2014 2014 2015 2015 2015 2015 2015 2016 2016 2016 2016 2016 2016	[32] [33] [57] [21] [35] [58] [38] [39] [20] [59] [41] [42] [44] [45] [29] [60] [48] [47] [22] [49] [19] [53] [9] [51] [54] [55]	Biochemical Biochemical Clinical Biochemical Biochemical Imaging Biochemical Biochemical Biochemical Clinical Autopsy Biochemical Biochemical N/M Biochemical Biochemical Biochemical Biochemical Autopsy Biochemical Biochemical Biochemical Biochemical Clinical Biochemical Biochemical	38 15 10 40 34 C/R 57 C/S 23 N/M ^a^ N/M 2 N/M 15 C/R 4 C/R C/R 8 N/M N/M 26 N/M 97 3	58 104 60 81 61 C/R 155 C/S 132 ^a^ ^a^ N/M 22 N/M 32 C/R 232 C/R C/R 19 N/M 110 48 66 563 221	65.5 14.4 16.6 49.0 55.9 C/R 36.7 C/S 17.4 ^a^ ^a^ N/M 9.0 30.0 46.8 C/R 1.7 C/R C/R 42.1 N/M 1.8 54.2 33.3 17.2 1.4
Cardiac involvement	1964 1977 2008 2008 2011 2011 2012 2012 2013 2013 2014 2014 2015 2015 2015 2015 2015 2015 2016 2016 2016 2016 2016 2016	[32] [61] [36] [62] [38] [20] [63] [41] [43] [44] [45] [29] [60] [64] [22] [48] [49] [50] [19] [9] [52] [53] [54] [55]	ECG Postmortem ECG, Imaging ECG, Imaging Imaging Imaging ECG, Imaging Autopsy ECG, Imaging ECG, Imaging N/M ECG, Imaging ECG, Imaging ECG, Imaging Autopsy ECG, Imaging Clinical ECG ECG, Imaging	9 C/S 7 C/R 11 71 8 ^a^ C/R 13 N/M 5 C/R C/R C/R 5 N/M N/M 37 6 C/S N/M 23 5	63 C/S 45 C/R 155 132 62 ^a^ C/R 22 N/M 32 C/R C/R C/R 232 19 N/M 45 48 C/S 110 563 221	14.3 C/S 15.5 C/R 7.1 53.7 12.9 ^a^ C/R 59.0 35.0 15.6 C/R C/R C/R 2.2 36.8 14.2 82.2 12.5 C/S 4.5 4.1 2.3
Lung involvement	1964 1977 2008 2008 2011 2011 2012 2013 2013 2015 2015 2015 2016 2016 2016 2016 2016	[32] [61] [36] [62] [39] [20] [41] [43] [44] [60] [22] [48] [19] [52] [55] [53] [54]	Clinical Postmortem Imaging Imaging Imaging Imaging Autopsy Imaging Imaging Imaging Autopsy Imaging Imaging Imaging Imaging Imaging Imaging	22 C/S 14 C/R C/S 60 ^a^ C/R 2 C/R C/R 6 N/M C/S 3 N/M 43	63 C/S 45 C/R C/S 132 ^a^ C/R 22 C/R C/R 232 N/M C/S 221 110 563	34.9 C/S 31.1 C/R C/S 45.4 ^a^ C/R 9.0 C/R C/R 2.6 N/M C/S 1.4 4.5 7.6
Neurological involvement	1964 1967 1974 2003 2008 2011 2011 2012 2014 2015 2016	[32] [57] [21] [58] [36] [38] [39] [41] [45] [64] [51]	Biochemical Clinical Clinical Biochemical Biochemical Clinical Clinical Autopsy N/M Clinical N/M	8 5 6 C/R 3 13 C/S ^a^ N/M C/R N/M	63 60 81 C/R 45 155 C/S ^a^ N/M C/R 66	12.7 8.3 7.4 C/R 6.6 8.3 C/S ^a^ 25.0 C/R 15.2
Pancreatic involvement	2008 2013 2016 2016	[37] [43] [52] [54]	Biochemical Biochemical Biochemical Biochemical	C/R C/R C/S 5	C/R C/R C/S 563	C/R C/R C/S 0.9
Bleeding manifestation	1959 1964 1966 1967 2011 2016	[12] [32] [33] [57] [39] [53]	Clinical Clinical Clinical Clinical Clinical Clinical	C/S 9 1 2 C/S N/M	C/S 63 104 60 C/S 110	C/S 14.3 0.09 3.3 C/S 30.9
Splenic involvement	1964 2011	[32] [59]	Clinical Clinical	2 N/M	63 ^a^	3.2 ^a^

*C/S* Case series, *C/R* Case report, *N/M* Not mentioned

^a^not possible to calculate

To estimate the proportion of patients with specific organ involvement, all case reports and case series with specific complications (e.g., case series with pancreatitis) were excluded. The reported rates varied widely among studies with renal involvement (median 48.7%), which was the most common complication followed by liver and cardiac involvement (Table 3).

**Table 3:** Reported percentages of organ involvement/complications among leptospirosis patients

Complication	No. of studies	Minimum %	Maximum %	Median
Renal	20	7.1	73.3	48.7
Liver	17	1.4	65.5	30.0
Cardiac	16	2.2	59.0	14.2
Pulmonary	8	1.4	45.4	8.3
Neurological	7	6.6	25.0	8.3
Haemorrhagic	4	0.09	30.9	8.8
Spleen	1	3.2	3.2	3.2
Pancreas	1	0.9	0.9	0.9

### Deaths, causes of death and case fatality rates based on individual studies

We identified 22 articles with reported causes of death among patients with leptospirosis. There were 4 articles from two databases, and we combined them for data synthesis. The final set included 5 case reports, 6 case series and 9 cross sectional studies. Table 4 summarizes the reported causes of death mentioned in leptospirosis-related publications in Sri Lanka. The exact cause of death according to ICD 10 was not mentioned in most of the articles. Hence, the causes of death are mentioned using the same wording in the article. Most major complications that can lead to death were reported from in Sri Lanka within the limited literature published since 1959.

**Table 4:** Reported deaths and reported causes of death

Published year	Study design	Deaths	Reported causes of death	Citation
1959	Case series	1	Haemorrhagic manifestation of liver and kidney	[12]
1964	Case series	2	Multi organ failure and coma (lungs, meningitis, neurological manifestations)	[32]
1967	Cross sectional	2	Leptospiraemic meningitis, bleeding manifestations	[57]
1974	Cross sectional	8	Sepsis with liver, renal manifestations	[21]
1976	Case report	9	Acute renal failure	[35]
1977	Case series	7	Cardiac and pulmonary manifestation (hypotension with or without tachycardia, pulmonary oedema, haemorrhages and exudations)	[61]
2008	Cross Sectional	15	Acute lung injury, myocarditis	[36]
2011	Cross Sectional	3	Fulminant myocarditis	[9, 38]^a^
2011	Cross Sectional	33	Respiratory failure and renal failure	[20]
2011	Case series and review	13	Multi organ failure	[39]
2012	Case series	21	Moderate to severe pulmonary haemorrhage in association with hepato-renal, myocardial and cerebral lesions	[41]
2013	Case control	1	Jaundice, oliguria with acute organ dysfunction	[42]
2014	Abstract	1	Meningoencephalitis	[45]
2015	Case report	1	Multi organ dysfunction and refractory shock, cardiac involvement (global hypokinesia, acute heart failure)	[60]
2015	Case report	3	Post-partum haemorrhage, HELLP syndrome	[23]
2015	Case report	1	Multi organ dysfunction syndrome	[47]
2015	Case report	1	Marked pleural effusion, cardiac, liver manifestations, renal manifestations	[22]
2015	Cross sectional	20	Hypotension, cardiac failure, AKI, ARDS,	[19]
2016	Cross sectional	7	Multi organ failure	[39, 58]^b^
2016	Case series	1	Cardiovascular, respiratory, abdominal complication, refractory hypotension, acute pancreatitis, multi organ failure	[52]

^a^References [9 and 28] used the same data set

^b^References [48 and 65] used the same data set

No publications assessed the mortality rates of Sri Lanka. However, we recruited studies with data on deaths after assessment for risk of bias. None of the articles reported the true mortality rate, as the sample selection was biased towards the objective of each particular study. Hence, the generalizability of death rates is limited.

Death rates were calculated considering the total number of probable cases of leptospirosis as the denominator. Probable cases of leptospirosis were defined as having positive results in either a screening or confirmatory test, including MAT, ELISA, Culture, PCR or any other serological tests. Table 5 summarizes the reported death rates in Sri Lanka.

**Table 5:** Reported death rates in Sri Lanka

Year	Study setting	Study population	Total no. of patients	Suspected	Confirmed	Deaths	Death rate by confirmed cases	Citation
1967	Rathnapura		60	0	60	2	3.33%	[57]
1974	Ragama		81	13	68	8	11.8%	[21]
2008	Colombo		45	0	45	15	33.3%	[36]
2011	Kandy, Mathale, Kegalle		401	246	155	3	1.9%	[38]
2011	Peradeniya		227	0	227	33	14.5%	[20]
2013	Colombo		40	40	40	1	2.5%	[42]
2014	ICU patients in selected hospitals		20	20	19	1	5.3%	[45]
2015	Kaluthara		45	0	45	20	44.4%	[19]
2016	Colombo, Homagama		232	0	232	7	3.0%	[58]^a^
2016	Colombo, Homagama		221	829	221	3	1.3%	[55]

^a^References 39 and 58 used the same data set

The highest death rates were reported from studies performed among Intensive care unit (ICU) patients. At General Hospital Kaluthara, 20 deaths out of 45 ICU patients confirmed for leptospirosis were reported, with a case fatality rate of 44.4% [19]. A study done among ICU patients at National Hospital of Sri Lanka reported a Case Fatality Rate (CFR) of 33.3%. Among usual ward settings, the highest death rate was from Teaching Hospital Peradeniya, with 33 deaths of 227 patients (CFR 14.5%) [20], followed by a study done in 1974 with a CFR of 11.7% [21]. However, in both these studies, there was no information on whether the investigators recruited all cases of leptospirosis through systematic patient enrolment. For CFR estimation, we included only three studies with more than 100 confirmed cases. The estimated pooled CFR was 7.0% (95% CI 5.2–9.4). Based on this CFR, the estimated annual death toll due to leptospirosis in Sri Lanka is approximately 730 (95% CI 542–980).

## Discussion

The estimated annual incidence of leptospirosis in Sri Lanka was 300·6 (95% CI 96·54–604·23) per 100,000 people. In our study, the incidence of leptospirosis, which requires hospital admission, was 52.12 (95% CI 51.69–52.57) per 100,000 people (Fig. 2). In the global burden of disease study, estimates were performed using a complex model that is valid for all countries, whereas our estimations were based on local data. Second, the data set available for Costa et al. [2]. included the two highest peaks, while our study included a larger time span, which might have diluted the estimation. The present estimates show that the number of deaths due to leptospirosis is significantly higher than the deaths due to dengue, which is considered the highest priority infectious disease in Sri Lanka. However, only 400 deaths were attributed to dengue, even during the largest outbreak in 2017, with nearly 200,000 reported cases, while we estimated an average of 730 deaths due to leptospirosis. Our review shows that the deaths were reported as a result of all the possible end organ involvement due to leptospirosis. Most patients died due to multi organ failure, even though kidney involvement was the most common. Among fatal cases, lung involvement was common. Leptospirosis deaths during pregnancy were also reported from Sri Lanka [22, 23]; however, the number of published studies is limited.

Calculating death rates based on scientific publications is challenging due to the difficulty of deciding the denominator. The denominator was defined as all confirmed cases of leptospirosis using any kind of investigation, including MAT, ELISA, PCR and culture techniques. In addition, some publications confirmed leptospirosis using other rapid detection methods, such as ELISA rapid kits and lateral flow immune assays [24, 25]. However, the validity of these techniques was questionable. Validity was assessed for most techniques compared to MAT. Although MAT was once considered the gold standard, it is no longer considered as such for various reasons [9]. Further, many studies from Sri Lanka used MAT, which was performed using the genus-specific Patoc strain without having a broad panel of serovars or regionally optimized serovars. Few articles reported use of broad panels. The strain of Leptospira isolated from Sri Lanka was previously published [26]. However, due to a lack of these cultures in local laboratories, these strains were not used in many studies. Culture is 100% predictive, although it is less sensitive due to difficulty in culturing. Hence, the reliability of the denominator is questionable. Nevertheless, culture-based studies have not been published in Sri Lanka since the 1970s.

The reported number of cases in IMMR was significantly higher than that in QEBs. IMMR does not include patients presented to the private sector, outpatient department or other complementary and alternative treatment modalities, whereas QEB should have all this information. However, QEB grossly underestimated the actual cases. The reason for this underestimate could be the lack of interest in reporting. In busy wards, many physicians do not consider notification a priority [27]. The medical statistics unit in the hospital will pick up all these cases and send them to the central statistics unit, and they will appear in IMMR. However, the same data will not be included in the QEB, which will only show notification data. Lack of familiarity with the case definitions, lack of knowledge among supportive staff, lack of interest in notifications and delay of notifications are the main deficiencies associated with the surveillance system of Sri Lanka [13, 28]. Urgent attention is required to correct this problem, which will have a major impact on disease control and resource allocation.

Our estimates are limited for several reasons. First, the private sector data and the outpatient department data were not included in both national databases. There were no studies on caseloads in these locations. In addition, the adjustment for underreporting was done using a single study due to the lack of other estimates. Both of these facts likely led to underestimation of the true caseload.

The diversity of clinical features in published research might be due to serovars in different geographical conditions and was described earlier as microgeographical changes of leptospirosis [29]. Even though serovar-specific clinical features are a major area of exploration, no proper attempts have been made to evaluate serovar-specific clinical features in the global literature. Large-scale culture isolation studies are required to assess serovar-specific complications. No culture-based publications have been reported from Sri Lanka since 1975 [30]. Deaths attributed to different serotypes can only be evaluated through prospective culture-based studies or newly developed genotyping studies, which are yet to be fine-tuned for direct patient samples.

Even though there may be publication bias, most of the studies reported leptospirosis outbreaks and increased case numbers in the wet zone. Areas with high precipitation appear to be at higher risk. Further, we observed that leptospirosis risk groups are moving beyond traditional occupational exposures within Sri Lanka. For example, outbreaks have been documented among people involved in ecotourism [31]. These findings may need specific public health preventive strategies.

## Conclusion

The Sri Lankan case study clearly shows the need for country-specific disease estimates using local data and to consider local factors affecting notification and surveillance. Even though the case numbers were lower than diseases such as dengue, the estimated case fatality rate of leptospirosis was more than 10 times of that of dengue. Further community-based studies on disease burden estimates are required to identify the true disease burden, and estimations of economic impact are required to observe the effect of this disease on the economies of individual countries.

## Additional files

Additional file 1: Quality assessment criteria. (DOCX 47 kb)

Additional file 2: Quality assessment of Sri Lankan leptospirosis research articles with morbidity estimates. (XLSX 40 kb)

Additional file 3: Quality assessment of Sri Lankan leptospirosis research articles with mortality estimates. (DOCX 72 kb)

## Abbreviations

AKI: Acute kidney injury
ARDS: Acute respiratory distress syndrome
C/R: Case Report
C/S: Case Series
CFR: Case Fatality Rate
CI: Confidant Interval
CMJ: Ceylon Medical Journal
DALY: Disability adjusted life years
ELISA: Enzyme linked immune sorbent assay
ICD: International classification of diseases
ICU: Intensive care unit
IMMR: Indoor mortality morbidity return
LERG: Leptospirosis burden epidemiology research group
MAT: Microscopic agglutination test
MODS: Multi organ dysfunction syndrome
MRI: Medical Research Institute
N/M: Not mentioned
PCR: Polymerase chain reaction
PGIM: Postgraduate institute of Medicine
QEB: Quarterly epidemiological bulletin
