# Supplementary material for: Estimating the burden of leptospirosis in Sri Lanka; a systematic review
Source: BMC Infect Dis. 2019 Feb 6;19:119. doi: 10.1186/s12879-018-3655-y (PMC6364467; doi:10.1186/s12879-018-3655-y)
Supplement: Supplementary file 1 — Quality assessment criteria. (DOCX 47 kb) [file 12879_2018_3655_MOESM1_ESM.docx]

**Table 1: Quality assessment checklist for case fatality estimation**

| **Criteria category** | **Quality** | | |
| --- | --- | --- | --- |
|  | **High:** The study fulfils all of the following criteria | **Medium:** The study does not fulfil criteria for high and low quality and in general has the following characteristics | **Low:** The study fulfils one or more of the following criteria |
| Study population | - All leptospirosis patients included (not from a specific subspecialty eg: intensive care unit/ nephrology unit : patient base recently and reliably estimated | - All leptospirosis patients included: patient base not recently or not reliably estimated | - Not all patients were included or data inadequate to assess |
| Disease confirmation | - Laboratory confirmation performed with standard methods and definitions, as defined by LERG - Active case ascertainment whether community or hospital/provider-based | - Laboratory confirmation performed but standard methods and definitions were not used. (Eg: MAT was performed, but not using a broad panel or regionally optimized panel) - Passive hospital/provider-based case ascertainment or not reported as active. | - Laboratory confirmation not performed. - Case ascertainment not performed as a systematic or continuous process |
| Biases | - Information available on proportion of possible, probable and confirmed cases for which single and paired sera were collected | - Paired sera collected, but information not available for proportion of possible, probable and confirmed cases | - No data on paired sera/ paired sera not collected |
| Analysis | - Rates calculated or can be extrapolated from the data | - Rates calculated or can be extrapolated from the data | - Rates cannot be calculated or extrapolated from the data |
