# Supplementary material for: Estimating the burden of leptospirosis in Sri Lanka; a systematic review
Source: BMC Infect Dis. 2019 Feb 6;19:119. doi: 10.1186/s12879-018-3655-y (PMC6364467; doi:10.1186/s12879-018-3655-y)
Supplement: Supplementary file 3 — Quality assessment of Sri Lankan leptospirosis research articles with mortality estimates. (DOCX 72 kb) [file 12879_2018_3655_MOESM3_ESM.docx]

Quality assessment checklist for mortality estimates.

| Serial | Year | Citation | Study ID | Study population | Disease confirmation | Lack of Biases | Analysis |
| --- | --- | --- | --- | --- | --- | --- | --- |
| 1 | 1967 | Thirivanavukkurasu et al | 49 | M | L | L | L |
| 2 | 1974 | Ramachandran et al | 22 | M | M | M | M |
| 3 | 2008 | Gunawardana et al | 24 | L | M | H | M |
| 4 | 2011 | Agampodi et al | 26 | H | H | H | H |
| 5 | 2011 | Kularathna et al | 28 | M | M | L | M |
| 6 | 2013 | Kalugalage et al | 31 | ? | M | M | ? |
| 7 | 2014 | Bandara et al | 34 | ? | M | L | ? |
| 8 | 2015 | Weeratunga etal | 42 | M | M | H | M |
| 9 | 2015 | Rajapakse et al | 39 | M | M | H | H |
| 10 | 2016 | Rajapakse et al | 58 | M | M | H | H |
| 11 | 2016 | Rajapakse et al | 47 | M | M | H | H |
|  |  |  |  |  |  |  |  |
|  |  |  |  |  |  | H-High |  |
|  |  |  |  |  |  | M-Medium |  |
|  |  |  |  |  |  | L-Low |  |
|  |  |  |  |  |  | ?- Data inadequate | |
